# Comparison between myocardial infarction with non-obstructive coronaries (MINOCA) and myocardial infarct patients with coronary artery disease (MI-CAD): A single-center retrospective cohort study

**DOI:** 10.22088/cjim.15.1.12

**Published:** 2024

**Authors:** Abbas Andishmand, Mahmood Emami Meybodi, Seyedeh Mahdieh Namayandeh, Hamid Reza Mohammadi, Mojtaba Andishmand, Mohammad Ali Zarbakhsh, Marzieh Azimi zade

**Affiliations:** 1 Yazd Cardiovascular Research Center, Non-communicable Diseases Research Institute, Shahid Sadoughi University of Medical Sciences, Yazd, Iran.; 2 Department of Epidemiology, Shahid Sadoughi University of Medical Sciences, Yazd, Iran.

**Keywords:** MINOCA, MI-CAD, STEMI

## Abstract

**Background:**

The coronary angiography results in a group of patients with myocardial infarction (MI) are normal or near-normal; which is diagnosed as myocardial infarction with non-obstructive coronary arteries (MINOCA). This study aimed to compare the mortality rate and risk factors between MINOCA and myocardial infarction with obstructive coronary artery (MI-CAD).

**Methods:**

This retrospective cohort study was conducted from January 1, 2018, to December 31, 2019. A total of 679 patients admitted to Afshar Hospital in Yazd with a diagnosis of ST-elevation myocardial infarction (STEMI) from 2018-2019 who underwent primary Percutaneous Coronary Intervention (PCI) were enrolled in the study. Demographic, and clinical variables, ECG finding and one-year mortality, were extracted using MI registry data from the Yazd Cardiac Research Center.

**Results:**

The estimated frequency of MINOCA was 4.6%. Patients with MINOCA (47.14±6.2) were younger than patients with MI-CAD (57.61±9.1) (P <0.0001). MINOCA patients (47.4±9.47) had a considerably greater left ventricular ejection fraction (LVEF) than MI-CAD patients (43.5±6.8) (P =0.018). The majority site of MI in MINOCA patients was located in the non-anterior wall (p <0.0001). A comparison of MINOCA and MI-CAD patients' one-year mortality revealed no significant difference (P =0.07).

**Conclusion:**

The prevalence of patients with MINOCA in Yazd was similar to other communities. Although these patients probably do not have a better prognosis, despite being younger and having better LV systolic function and lower CAD risk factors.

The pathogenesis of ST-elevation myocardial infarction (STEMI) is typically a coronary artery occlusion (1). While it is possible that in some patients, the severity of vascular stenosis is not high (2). Myocardial Infarction with Non-obstructive Coronary Arteries (MINOCA) is defined as STEMI which coronary angiography is normal or near-normal (less than 50% stenosis) (3). Numerous significant investigations have revealed that MINOCA is prevalent in 5-6% (range 5-15%) of patients with acute myocardial infarction (MI) and that its frequency varies in populations (4-7). MINOCA is an important problem in the diagnosis and treatment which has several causes, so extensive diagnostic tests are needed to find its etiology. MINOCA has ischemic and non-ischemic causes. Ischemic etiologies include the epicardial or microvascular spasm, spontaneous coronary artery dissection, plaque rupture, microvascular, and thromboembolism. Its non-ischemic etiologies are myocarditis, and Takotsubo syndrome (8-10). 

In ESC guidelines of 2017, MINOCA is included in defining the MI (11). The clinical management of patients with MINOCA and Myocardial infarction with obstructive coronary artery (MI-CAD) has differences, and knowing more about the differences between these two groups can help clarify the appropriate diagnostic and treatment methods (12, 13). Recent studies have also looked at the variations between these two categories' characteristics to improve patient care and survival in the years following myocardial infarction, although more research is still required (14, 15). This study was conducted because of the lack of statistics on patients with MINOCA in the center of Iran and the demographic differences between Iran's population, and other countries. This study aimed to compare the mortality rate and risk factors between MINOCA and MI-CAD.

## Methods

This retrospective cohort study was conducted at Afshar Hospital affiliated with Shahid Sadoughi University of Medical Sciences in Yazd, from January 2018 to December 2019. This study was performed on 679 patients with STEMI candidates for primary Percutaneous Coronary Intervention (PCI) in Afshar Hospital. STEMI according to criteria provided by American College of Cardiology (ACC) and European Society of Cardiology, is defined as having a 24- hour of chest pain onset associated with an increase in troponin and ST-segment elevation measured from J point (≥0.2 mV in V1-V3 or ≥0.1 mV in other leads) (16, 17). 

They were incorporated into the study using the simple sampling technique. The study excluded participants with contraindications for angiography, those with a history of at least one of MI, PCI, CABG, NSTEMI, or thrombolytic treatment, as well as those with diagnoses other than MI. Eligible participants with STEMI diagnosis who met the inclusion criteria suitable for angiography, and coronary interventions underwent coronary angiography via femoral artery access. After the primary PCI, the patients received medical treatment based on the routine instructions of Afshar Hospital and were monitored for 24 hours in the hospital. Two cardiologists saw all coronary angiographies, and if there was a normal angiography or stenosis less than 50%, the patient was diagnosed with MINOCA (3, 18). The patients were followed up for one year, and demographic, clinical, cardiac death frequency, and imaging finding were extracted using MI registry data from Yazd Cardiac Research Center. For data analysis, Version 23 of SPSS program (SPSS Inc., Chicago, USA) was used. Continuous variables were expressed by mean ± standard deviation, whereas categorical variables were expressed by numbers (percentages). Chi-square test was used for categorical variables, while the t-test was utilized for continuous variables. The statistical significance level was below 0.05. The study was conducted with the agreement of Ethics Committee of Yazd Islamic Azad Medical School.

## Results

679 patients with STEMI underwent coronary angiography interventions. 25 patients who had the final diagnosis of pericarditis (17 cases), myocarditis (3 cases), pulmonary embolism (1 case), and early repolarization (4 cases) were excluded from the study. A total of 29 (4.6%) individuals with a diagnosis of MINOCA were compared with 625 (95.4%) patients who had MI-CAD. The patients with MINOCA were younger and with a lower BMI than the group with MI-CAD. The comparison of comorbidities between two groups showed that hypertension was significantly more frequent in MI-CAD group.Left ventricular ejection fraction (LVEF) in the MINOCA group (47.4±9.47) was significantly higher (P=0.02) than MI-CAD group (43.5±6.8). 

The incidence of anterior myocardial infarction in the MINOCA group (20.6%) was significantly lower (p<0.001) than in MI-CAD group (57.8%). In the present study, only 55 (8.8%) cases of cardiovascular and non-cardiovascular deaths were reported in the MI-CAD group, and there was no death in the MINOCA group (P=0.07) (table 1). 

**Table 1:** Comparison of the MINOCA and the MI-CAD patients

**Variable**	**MINOCA** **(N=29)**	**MI-CAD** **(N=625)**	**P-value**
**Age (y), Mean±SD**	47.14 ± 6.2	57.61 ± 9.1	**<0.001** ^ a^
**BMI (kg/m2), Mean±SD**	25.3 ± 3.5	26.5 ± 4.04	**0.04** ^ a^
**Sex (male), N (%)**	22 (76.7)	423 (67.7)	0.08 ^b^
**DM, N (%)**	5 (17)	202 (32.3)	0.09 ^b^
**HTN, N (%)**	8 (28)	318 (50.8)	**0.005 ** ^b^
**Smoking, N (%)**	10 (34)	231 (36.9)	0.45 ^b^
**HLP, N (%)**	10 (34)	211 (33.8)	0.5 ^b^
**LVEF (%), Mean±SD**	47.4 ± 9.47	43.5 ± 6.8	**0.02** ^ a^
**Site of myocardial infarction, N (%)**			**<0.001** ^b^
**Anterior**	6 (20.7)	361 (57.8)	
**Non-anterior**	23 (79.3)	264 (42.2)	
**One-year mortality, N (%)**	0	55 (8.8)	0.07 ^b^

MINOCA myocardial infarction without obstructive coronary artery, MI-CAD myocardial infarction with obstructive coronary artery. DM diabetes mellitus, HTN hypertension, HLP hyperlipidemia, BMI body mass index, LVEF left ventricular ejection fraction. Data are expressed as mean ± SD, or number (percent).^ a;^ independent t test, ^b ^: chi-square test.

## Discussion

In all countries, acute myocardial infarction is the major cause of mortality, mainly attributable to thrombotic obstruction of the coronary arteries. This may occur as a separate entity called MINOCA where there is no obstructive coronary lesion. In this research conducted just on STEMI patients, we discovered a prevalence of 4.6%. While most studies have included both STEMI and NSTEMI patients, we have exclusively examined STEMI patients. In our study, patient selection was strict because there is less error in STEMI diagnosis than in NSTEMI diagnosis and the differential diagnosis of NSTEMI is broader. Electrocardiographic changes similar to those seen in NSTEMI occur in many non-coronary heart diseases, and non-cardiac diseases and using many medications. Besides, an abnormal increase in troponin, which indicates myocardial damage, is seen in non-coronary heart disease and many systemic diseases.

 In contrast, NSTEMI patients have coronary angiography later, and early medical therapy, particularly anti-platelet and anti-coagulation medicines, might induce the coronary artery to open prior to angiography and influence the occurrence of MINOCA. Therefore, the results of our study can be affected by this strict selection. However, the prevalence of 4.6% was comparable to other studies. As mentioned earlier, MINOCA has a different population prevalence, part of which was in terms of heterogeneity and non-selectivity. In the study of Nordenskjöld et al. on MI patients registered with the SWEDEHEART registry, the prevalence of MINOCA was approximately 4.6% (19). 

The prevalence of patients with MINOCA was 6.2% in Turkey (20). The prevalence of MINOCA in South Korea, was about 4.4%. (21) In Canada, the prevalence of MINOCA was 8.2% (6). In the study of Pasupathy et al. was considered that based on the studies the prevalence of MINOCA was 6% (13). In our study, the age of MINOCA patients was significantly lower than that of MI-CAD patients. The results of other studies were similar (4, 13, 20). It is clear that coronary artery atherosclerosis is age-related, and its prevalence and extent increase with age (22). On the other hand, increasing age is associated with a high risk of death after MI (23). In our research, the MINOCA group had greater LVEF, one of the most popular, practical, and accepted metrics for evaluating left ventricular systolic function. Decreased LVEF after myocardial infarction was independently related to an increase in sudden cardiac death (24). The prevalence of important CAD risk factors, such as hypertension, was significantly lower in MINOCA patients. Hypertension, short-term and long-term, is associated with an increase in fatal cardiac events following MI (25). Diabetes mellitus increases mortality after MI, especially in women, but no significant difference was found in our study (26). There was a substantial difference in BMI between two groups. Except for diabetic individuals, studies demonstrate an inverse association between BMI and the risk of mortality from a MI (27, 28). A one-year follow-up of patients was performed. During this period, no cardiac death occurred in MINOCA patients, while 8.8% of MI-CAD patients had cardiac death, but it is not statistically significant, this suggested that MINOCA patients may not have a better prognosis than MI-CAD patients. Findings on the prognosis of MINOCA in comparison to MI-CAD have been inconsistent; some studies have shown a more positive prognosis, while others have revealed no appreciable difference between the two groups. ACTION Registry-GWTG study indicated that MINOCA had significantly lower in-hospital mortality than MI-CAD (1.1% vs. 2.9%, respectively) (23). 

Pelliccia et al.’s found that the prognosis in MINOCA was slightly better than in MI-CAD. At a median follow-up of 25-month, 3.8% of deaths occurred in MINOCA group. At the same time, beta-blocker consumption and ST depression related to worse outcomes (29). In Choo et al.’s study showed that two-year mortality of MINOCA patients is comparable to that of MI-CAD patients (9.1% and 8.8%, respectively) (30). Although MINOCA cases were relatively rare, the findings show similarities with other communities. However, younger age, lower prevalence of hypertension, and better LVEF did not improve prognosis. Studies showed that MINOCA was more common in women (31). Nevertheless, our findings were the opposite. The reason that our patients were mostly male was in terms of the limited selection of sample. First, the male-to-female ratio is higher in STEMI than in NSTEMI. Second, microvascular disorders are more common in women despite normal angiography of epicardial coronary arteries. Third, coronary angiography is not as common in women as it is in males. Patients with MINOCA are unexpectedly less likely to obtain medication (32). For example, one study found that only 50% of patients were prescribed dual anti-platelet drugs, which can lead to recurrent myocardial infarction (33). 

First, the sample size of this study was small, and therefore, the prognosis of MINOCA patients cannot be accurately judged. Second, the findings were not generalizable to other ACS cases because sample selection was exclusively performed on STEMI patients. A more comprehensive study with a larger scale and longer follow-up are suggested.  We can conclude from this study that the prevalence of MINOCA in Yazd is almost similar to other communities. However, these patients do not have a better prognosis despite being younger and having better LV systolic function and lower CAD risk factors.
